# Learning from the challenges of undertaking an evaluation of a multi-partner housing support initiative delivered within a hospital setting

**DOI:** 10.1016/j.puhip.2022.100333

**Published:** 2022-10-31

**Authors:** A. Foster, E. Holding, E. Lumley, S. Roxby, D. Portman, J. Holliday, A. Peace, I. Del Rosario, W. Khan, A. Brenman, M. Gillett, E. Goyder

**Affiliations:** a School of Health and Related Research, University of Sheffield, Sheffield, UK; b Wakefield District Housing, Wakefield, UK; c Mid-Yorkshire NHS Trust, Yorkshire, UK; d School of Health and Related Research, University of Sheffield (Formerly at South West Yorkshire Partnership NHS Foundation Trust), Yorkshire, UK; e South West Yorkshire Partnership NHS Foundation Trust, Yorkshire, UK

## Abstract

**Objectives:**

We present learning from a mixed-methods evaluation of a housing support initiative for hospital inpatients.

**Study design:**

A mixed-methods process evaluation.

**Methods:**

A social housing provider delivered a housing support service in two hospitals (mental health unit and general hospital). Healthcare providers, the social housing provider and academic researchers designed and undertook a co-produced, mixed-methods process evaluation of the intervention. The evaluation included questionnaires, semi-structured interviews, analysis of routinely collected data and economic analysis. Despite commitment from the partners, the evaluation faced challenges. We reflect on the lessons learnt within our discussion paper.

**Results:**

Despite the commitment of the partners, we faced several challenges.

We took an iterative approach to the design and processes of the evaluation to respond to arising challenges. Recruitment of service-users was more difficult than anticipated, requiring additional staff resources. Given the small-scale nature of the intervention, and the quality of data recorded in hospital records, the planned economic analysis was not feasible. Positive factors facilitating evaluation included involvement of staff delivering the intervention, as well as managers. Being able to offer payment to partner organisations for staff time also facilitated ongoing engagement.

**Conclusions:**

Multi-partner evaluations are useful, however, researchers and partners need to be prepared to take an iterative, resource intensive approach. Both availability and quality of routine data, and the resources required to support data collection, may limit feasibility of specific methods when evaluating small-scale cross-sector initiatives. Thus, this necessitates a flexible approach to design and analysis.

## 1 Introduction

Healthcare services are increasingly supporting service-users with addressing social determinant of health issues including financial problems, inappropriate housing and food insecurity [1]. To manage these needs, healthcare services are working with voluntary and community sector organisations (VCSEs) such as charities and social housing providers to provide specialist support [2]. For example, within the United Kingdom, debt advice workers are based within primary care services. Initiatives are often developed in one locality as short-term, pilot projects [3]. Evaluations may be undertaken by university-based researchers in conjunction with healthcare and VCSE partners to understand intervention delivery, impact and to inform future funding decisions [4]. However, despite increased impetus to undertake and evaluate complex cross-sector interventions, multi-partner evaluations can face challenges. This is partly due to the scale and nature of the interventions. In this short report, we reflect on our experience of undertaking a multi-partner evaluation of a hospital-based housing support initiative. Our learning will be useful for other researchers, along with health and VCSE partners planning similar evaluations.

### 1.1 About the intervention and evaluation

A housing support intervention was delivered by a social housing provider in two hospitals in Yorkshire, England (a general, district hospital and a mental health unit). It involved two specialist housing officers (one based in each hospital) working with hospital inpatients to address their housing issues to facilitate discharge. Examples include organising accommodation for people experiencing homelessness and arranging financial support so people can remain in their home [5]. Healthcare managers, the social housing provider and commissioners worked with researchers from the University of Sheffield to co-design and undertake a mixed-methods evaluation between 2019 and 2022. The aim was to understand delivery mechanisms and the intervention's impact on individuals and the healthcare system. The evaluation consisted of:

• A service-user questionnaire (n = 37)
• Qualitative interviews with service-users and hospital and housing staff (n = 16)
• Secondary analysis of routinely collected data from the social housing provider (n = 488)
• We were unable to conduct the planned economic analysis utilising routinely collected data from the social housing provider and the hospitals. However, we did undertake some threshold analysis.

A description of the evaluation's methods and findings are reported in Ref. [5]. Our evaluation was disrupted by Covid-19, however, we are not discussing this because the impact of Covid-19 on research has been explored elsewhere [6].

## 2 Findings

### 2.1 Challenges encountered and solutions identified

Within this discussion paper, we describe how we had to take an iterative approach to the design and processes of the evaluation to respond to arising challenges. This included giving service-users support to participate in the study which required additional staff resource. The small-scale nature of the intervention and the quality of data recorded in electronic hospital patient records made it problematic to undertake some research methods, especially economic analysis. This indicates that some methods may not be feasible when evaluating smaller-scale interventions. Ensuring healthcare and housing partners are sufficiently renumerated for research activities may facilitate engagement.

### 2.2 Taking an multi-partner, iterative approach to the evaluation design and processes

The healthcare, housing, and research partners worked together to develop and adapt the evaluation's design and processes in response to challenges. For example, it was difficult to recruit participants through the hospitals. Instead, the social housing provider recruited interviewee participants and we were able to utilize the social housing provider's routinely collected service-user data to supplement the low response rates to the questionnaire. Teams need to anticipate taking an iterative approach to an evaluation's design. However, taking an iterative approach can have ethical implications and cause delays. We had to pause the study at times whilst undertaking ethical amendments due to making changes to the evaluation processes. Whilst it is imperative that studies have ethical approval, current approval processes are not conducive to undertaking evaluations of complex interventions. For example, it may be more efficient but not generate additional risk if researchers are able to make small changes to study processes without having to pause the evaluation.

The multi-partner approach was beneficial however, partners' were not sufficiently renumerated and the housing support officers delivering the intervention were not adequality involved in the evaluation. Much of the project relied on the goodwill of partners contributing their time to the evaluation without receiving renumeration for the time they spent on research. We found that committing some financial resource at points facilitated engagement. For example, the study paid the hospital for 3 days of a data manager's time to extract information from the hospital patient records. Whilst only a small amount of funding, it was an important gesture. Given staffing pressures within healthcare and VCSE organisations, there is a need for studies to adequately cost in different partners' time rather than purely funding the academic researchers.

A further challenge was ensuring that people delivering the intervention, not just their managers, were sufficiently involved in the evaluation. We found that when we involved the housing support officers, they were able to provide useful information including on participant recruitment methods and on interpreting the findings. However, there were some gate-keeping issues to their involvement along with methodological challenges of involving the people delivering the intervention in the evaluation. Participatory research methods could support evaluation teams with managing these issues.

### 2.3 Challenges to recruitment and data collection when interventions are small-scale, and service-users find it difficult to engage

We found that some research methods may need greater staff resources, or may not be feasible to use, when evaluating locally-based interventions due to the relatively small service-user population. Many of the service-users had complex lives and needed considerable support to participate. Furthermore, there was also higher than anticipated rates of attrition. For the qualitative interviews, the evaluation team invested significant periods of time in organising and undertaking interviews. This required additional staff time and needs to be factored in when resourcing evaluations. Experienced qualitative researchers were required because the interviews were challenging to conduct due to the vulnerable nature of the population. For the questionnaires, we struggled to collect follow-up responses. People were either too unwell or were no longer willing to engage. Our experience of higher rates of attrition indicates that future evaluations may want to over-recruit participants, albeit this requires additional resource.

The smaller-scale nature of the intervention had implications on suitable evaluation methods. The housing intervention consisted of two housing officers supporting approximately 250 people per year. Consequently, there was a relatively small number of people to recruit to the evaluation. For example, allowing six months for recruitment to the questionnaire with a response rate of 50% would only yield a sample size of 50. In hindsight, we should have had more detailed conversations with the housing providers about caseloads and how this size impacts on the feasibility of certain quantitative methods. Furthermore, the smaller caseloads mean that longer participant recruitment periods are required. We found that the resources and time required for the questionnaire was not justified given the number of responses we received, indicating that other methods may be more feasible.

Rather than using questionnaires, we could have made better use of the routine data collected by the social housing provider. The provider recorded data on each service-user such as their demographics and housing outcomes. On reflection, we could have expanded this routine data set such as to include Patient Reported Outcome Measures. When designing future evaluations, we recommend exploring the feasibility of expanding the variables already being collected through routinely collected data processes.

### 2.4 Capturing impact on the healthcare system

It can be difficult to capture impact on healthcare utilisation and other healthcare related impacts may be more relevant. A key motivation for funding the housing support service was to decrease healthcare utilisation, specifically reducing the length of hospital inpatient admissions. However, it was problematic to capture changes in service use because of omissions within the hospital electronic patient record systems. This indicates that researchers need to consider methods such as healthcare utilisation questionnaires and invest time in supporting healthcare staff to improve the quality of data capture. For example, by delivering training to ward staff about how the data will be used to inform service investment. Furthermore, our evaluation identified that the housing support intervention appeared to deliver benefits to the healthcare system beyond individual service use. In the qualitative interviews, healthcare staff reported spending considerably less time on housing issues, which freed up time to spend on clinical tasks. However, we did not quantitatively capture this information. It is recommended that future evaluations draw upon innovative economic analysis methods to capture healthcare service impact including staff time spent on non-medical needs.

## 3 Conclusion

Multi-partner evaluations are valuable in influencing practice and policy but need to be flexible, adapting methods and processes to respond to arising challenges. This approach is resource intensive, and studies should consider renumerating non-academic partners for their involvement. Greater consideration is needed about which quantitative methods are feasible when evaluating smaller-scale interventions especially in terms of capturing outcomes and service utilisation data.

## Ethical statement

Ethical approval for this project was granted by North of Scotland Research Ethics committee and the Health Research Authority (REC reference: 20/NS/0050) (4th May 2020).

## Funding statement

This project is funded by the National Institute for Health and Care Research (NIHR) School for Public Health Research (SPHR) (Grant reference PD-SPH-2015). The views expressed are those of the authors and not necessarily those of the NIHR or the Department of Health and Social Care.

## Declaration of competing interest

None of the authors have any conflicts of interest.
